# Do the Powerful Discount the Future Less? The Effects of Power on Temporal Discounting

**DOI:** 10.3389/fpsyg.2017.01007

**Published:** 2017-06-21

**Authors:** Jinyun Duan, Sherry J. Wu, Luying Sun

**Affiliations:** ^1^Department of Psychology, Soochow UniversitySuzhou, China; ^2^Department of Psychology, Princeton University, PrincetonNJ, United States

**Keywords:** power, temporal discounting, optimism, experience of frustration, *Danbo*, cross-cultural

## Abstract

Individuals have the tendency to discount rewards in the future, known as temporal discounting, and we find that sense of power (the felt capacity to influence the thinking and behavior of others) reduces such tendency. In Studies 1 and 2, we used both an experiment and a survey with organizational employees to demonstrate that power reduced temporal discounting. In Study 3, we replicated study 1 while exploring a unique cultural trait of *Danbo*, or indifference to fame and wealth, across two ethnic groups (Han and Tibetan groups) in China. While power reduces temporal discounting, the relationship between the two may be leveraged by individual differences of optimism, frustration, and *Danbo*. The results imply a more nuanced interpretation of how individual and situational factors can affect intertemporal choice.

## Introduction

Individuals make choices and decisions by comparing costs and benefits at various points in time, for instance, a choice of $100 now and $110 in a week, consumption and deposit, and pleasure of smoking at the moment and health in the future. By weighting and trading off costs and benefits at different points in time, individuals make a corresponding decision of what and how much resource to take or allocate across time. This is known as intertemporal choice (Loewenstein, 1988; Loewenstein and Thaler, 1992). Researchers explain much of individuals’ behavioral disparity in intertemporal choices by temporal discounting (Kirby and Marakovic, 1995; Green and Myerson, 2004; Doyle, 2013).

Temporal discounting refers to an individual’s tendency to perceive a desired result in the future as less valuable than one in the present, which is also known as time discounting or delay discounting (Rodzon et al., 2011). Temporal discounting is an important consideration for research in intertemporal choice. According to previous research, individuals assign relative values to different payoffs at various points in time and they tend to give greater value to payoffs as they move closer to the present moment. For example, individuals prefer to receive $100 now rather than receive $150 in 1 year.

Power refers to the control of important resource such as information and money (Fast et al., 2009, 2012; Dubois et al., 2010; Maner et al., 2012), or the ability to influence others’ thinking and behavior (Keltner et al., 2003). The association between power and decision-making behavior has been investigated extensively. For example, Anderson and Galinsky (2006) found that high-power individuals tend to be more optimistic when evaluating potential risks so that they are more risk-taking. In addition, See et al. (2011) suggested that compared with people with a low level of power, people with a high level of power are more likely to make biased decisions. However, the relationship between temporal discounting, which significantly influences intertemporal choice, and power is less investigated (Joshi and Fast, 2013). Therefore, the aim of the current study is to investigate how power influences temporal discounting, including the boundary conditions if such a relationship holds.

The current research provides novel theoretical insights to the existing literature in several perspectives. Firstly, it provides a new perspective for studies on temporal discounting and intertemporal choice. Prior research mainly investigated temporal discounting from the attributes of decision-making options, including the relative value of choice options and the length of time delay (Read et al., 2005), or from the situational factors where a choice option might occur and have impact on, such as from factors in finance, health or social policies (Hardisty and Weber, 2009). However, characteristics of decision-makers, such as individual differences of personality, and motivations and construals, are relatively uninvestigated in previous research. The present study focuses on individuals’ perceived sense of power as an influencer of temporal discounting, and explored the mechanisms and boundary conditions of such a relationship. Secondly, the current research will enrich our knowledge on the cognitive effects of power (Duan and Huang, 2013; Duan et al., 2015), and in particular extend the application of the approach-avoidance theory of power, which claims that elevated power is associated with increased rewards and activates approach-related tendencies (Keltner et al., 2003). Thirdly, this study explores a culturally unique feasure called *Danbo* and its moderating effect on the relationship between power and temporal discounting. *Danbo, or* [scale=.50]img001 as in Chinese, refers to a life attitude of being indifferent to fame and wealth. Wang and Cui (2004) suggested that *Danbo* is a unique cultural trait which belongs to a certain set of East Asian cultures. Therefore, the current research explores cross cultural differences by localizing research of temporal discounting in different ethnic groups in China. Finally, the results of this study may provide insights for intertemporal choice and decision-making in management practices.

## Hypotheses

### The Effect of Power on Temporal Discounting

The construal level theory in social psychology delineates the relation between psychological distance and the extent to which people’s perception about an object or event is concrete or abstract, or as low or high level (Trope and Liberman, 2003). High level construal is when people think abstractly, while low level construal is when people think more concretely and is associated with psychological proximity. In the task of intertemporal choice, amount of money tends to be regarded as a high construal level attribute, while time tends to be regarded as a low construal level attribute. The reason why individuals tend to give up relatively large but delayed reward and prefer relatively small but instant reward is that they pay more attention to the time, the low construal level attribute, rather than amount of money, the high construal level attribute (Trope and Liberman, 2003). From this perspective, we predict that sense of power will decrease the tendency of temporal discounting. There are three reasons for this prediction. Firstly, Magee and Smith (2013) suggested that people with a high sense of power tend to focus on high construal level attributes, so that they will focus on the amount of money instead of the length of time. Their evaluation of rewards decreases more slowly as they approach a temporal horizon in the future and they are more likely to wait and take risks. Secondly, individuals with a higher sense of power have a stronger feeling of controlling their future, and the distance between their present self and future self is closer (Kanten, 2011). Since one of the factors which cause temporal discounting is an uncertain feeling of future (Frederick et al., 2002), if individuals feel that they are unable to control their future or they are not sure whether they can get reward in the future, they are more likely to give up delayed gratification and choose instant rewards (Ersner-Hershfield et al., 2009; Bartels and Rips, 2010). Thirdly, according to prospect theory (Kahneman and Tversky, 1979), giving up instant gratification is perceived as loss. Individuals tend to avoid loss, so that they are unwilling to give up instant gratification and wait delayed gratification, which leads to temporal discounting. Keltner et al. (2003) suggested that people with a high sense of power tend to focus on obtaining benefit and reward when making a decision, while those with a low sense of power tend to focus on avoiding loss in decision making. Therefore, compared with people with a low sense of power, people with a high sense of power are less sensitive to the perceived “loss” from giving up instant gratification so that they will experience less temporal discounting. Considering what mentioned above, we propose Hypothesis 1.

**H1: Sense of power will decrease the tendency of temporal discounting. High power individuals are less likely to engage in temporal discounting than low power individuals.**

### The Mediating Effect of Optimism

Previous research indicated that an individual’s tendency of temporal discounting is not only associated with the amount of money and the length of time, but also the risk perceived by the individual (Green and Myerson, 2004). If people have to choose between ‘get $100 now’ and ‘get $120 in 1 year,’ they may consider the reward from the latter choice as less certain and riskier than that from the former choice because there is a possibility that they may not get the benefit in a year (Frederick et al., 2002). However, if individuals are optimistic when facing risks and uncertainties, the tendency of temporal discounting may decrease as they remain relatively immune to risks.

According to the approach-avoidance theory of power (Keltner et al., 2003), people with a high sense of power tend to focus on rewards so that they will engage in more approach behavior. On contrast, people with a low sense of power are sensitive to risks and their behaviors are more constrained in various situations, so that they will engage in more avoidance behavior. Compared low power individuals, high power individuals are more optimistic and confident when evaluating risks since they perceive themselves as having more resources and capability to cope with risks. In addition, power will increase the experience of positive affect and raise optimism further (Galinsky et al., 2003; Anderson and Galinsky, 2006; Levine, 2010). Thus, facing intertemporal choice, individuals with a high sense of power may be more optimistic evaluating risks in uncertainty and their risk compensation is relatively low, so that their tendency of temporal discounting may be lower. Considering what mentioned above, we propose Hypothesis 2^1^.

**H2: Optimism will mediate the relationship between power and temporal discounting.**

### The Moderating Effect of Frustration Experience

The positive relationship between power and optimism may be influenced by many other situational factors (Chen et al., 2001). In this study, we predict that frustration experience will moderate the relationship between power and optimism.

Firstly, in terms of intertemporal choice, high power individuals are more optimistic so that they are also more risk seeking, more attentive to future rewards, and less sensitive to uncertainties (Anderson and Galinsky, 2006); but only when frustration is absent. We predict that when primed with frustration experience, high power individuals will tend to avoid risks as low power individuals do, as remembering failure of coping with risks they have experienced before decreases their optimism and leads to instant gratification rather than delayed gratification. Secondly, when high power individuals feel frustrated, they may be more sensitive to threats in the surroundings and perceive the current situations as less stable; i.e., they may lose their power at any time. Such perception decreases level of optimism (Lammers et al., 2008). Therefore, in intertemporal choice, they may be less willing to wait and prefer instant gratification rather than delayed gratification. When the powerful feel like they may lose their power at any time, accepting instant rewards may be a good strategy. While optimism may decrease the tendency of temporal discounting as proposed in H1 and H2, the mediated effect of optimism on temporal discounting may be moderated by frustration experience. Thus, we propose Hypothesis 3a and Hypothesis 3b.

**H3a: Frustration experience will negatively moderate the relationship between power and optimism. In other words, frustration experience will decrease the strength of the positive relationship between power and optimism.**
**H3b: The interaction between power and frustration experience will be mediated by optimism, which makes up a mediated moderation model.**

### The Moderating Effect of *Danbo* Trait

Power increases the level of optimism and subsequently decreases the tendency of temporal discounting. However, the mediating effect of optimism is not always effective. Apart from frustration experience, we predict that *Danbo* trait (an East-Asian cultural value of being indifferent to fame and wealth) will moderate the relationship between power, level of optimism, and temporal discounting, for the following reasons.

Firstly, Van den Bergh et al. (2008) found that activating the general reward system, such as exposure to monetary or erotic stimuli, leads to more impatience in intertemporal choice. Mittal et al. (2013) found that greedy individuals have lower self-control capacity and more impulsive behavior, which makes it harder for them to resist the temptation of immediate rewards. However, high *Danbo* goes parallel with low materialistic desire and a less sensitive reward circuitry, even when the sense of power is low. Therefore, we predict that high *Danbo* individuals are more willing to delay gratification and have lower time discount tendencies, regardless of their sense of power. Secondly, Loewenstein (1988) found that one explanation of temporal discounting is loss aversion. Since people with a low power are more likely to focus on avoiding loss when they make decisions (Keltner et al., 2003), we predict they have a higher tendency to discount the time, but only when the low power individual has a low *Danbo* trait like most of the people who gain utility value from materialistic reward. When people have a high *Danbo* trait regardless of power, their desire for materialistic reward is defined to be low (Wang and Cui, 2004). The same amount of loss looms smaller for high *Danbo* individuals and they should experience less loss aversion. As a result, tendencies to time discounting will be reduced. In summary, we predict that for high *Danbo* individuals regardless of power, they require less risk compensation, and have a lower tendency of time discounting; for most of us who do not have a high *Danbo* trait, lack of power is associated with being less optimistic to deal with uncertainties during waiting, higher risk compensation, and a higher tendency of time discounting. Combined with H1 and H2, H4 was proposed.

**H4: The relationship chain “power — optimism — temporal discounting” will be established only in the case that the individual has a low *Danbo* trait.**

## Study 1: The Influence of Power on Temporal Discounting

### Method

#### Participants

A total of 84 undergraduate students participated in this study. Three of them did not finished the recall task and two of them had more than two indifference points^2^ in the tasks of temporal discounting, so that these five participants were not included in data analysis. In addition, another one participant was excluded because his rate of temporal discounting *k* is zero (between the choice of “get ¥120 at once” and the choice of “get ¥120 in 1 year”, he chose the latter one)^3^. Hardisty and Weber (2009) indicated that *k* ≤ 0 is not consistent with the perspective of rational decision making, because it indicates that the decision maker prefers to receiving gains later rather than now, or prefers to suffering losses now rather than later. The final sample of participants was composed of 41 male undergraduate students (52.56%) and 37 female undergraduate students (47.44%). The range of age is from 18 to 23 years old (*M* = 20.44, *SD* = 0.98).

#### Design and Procedure

##### Power mind-set manipulation

Participants were randomly assigned into two conditions: *high-power* (*n* = 39) and *low-power* (*n* = 39). The power mind-set manipulation was adapted from the experiential power prime (Galinsky et al., 2003). Participants were asked to write a narrative essay about a particular incident in their lives after reading the definition of power (“as a situation in which you controlled the ability of another person or persons to get something you wanted, or were in a position to evaluate those individuals”). Participants in the *high-power condition* were asked to recall a particular incident “in which you had power over another individual or individuals” and their feelings at that time, whereas participants in the *low-power condition* were asked to recall a particular incident “in which someone else had power over you.”

##### Temporal discounting task

After answering distractor questions unrelated to the study, participants were instructed to make a series of intertemporal choices in which they chose between immediate and future outcomes (Hardisty and Weber, 2009). A scenario was first presented to the participants: “Imagine you just won a lottery, worth ¥120, which will be paid to you immediately. However, the lottery commission is giving you the option of receiving a different amount, paid to you 1 year from now.” Participants answered 10 binary choice questions where they chose between receiving ¥120 or receiving a different amount 1 year in the future. This procedure was used to elicit the indifference point where participants were indifferent between present and future gains. The calculation formula is *k* = (*A*/*V*-1), where *k* represents the rate of temporal discounting, *A* represents delayed gratification, and *V* represents instant gratification. The bigger *k* is, the greater tendency of individuals to experience temporal discounting will be.

Optimism measure (adapted from Anderson and Galinsky, 2006). Participants answered three questions with a 7-point Likert scale (1 = disagree strongly to 7 = agree strongly), including “Although there is the risk of delayed reward, I have the ability to deal with it,” “I am optimistic with the chosen reward in 1 year” and “I do not think that there is an unexpected situation where I cannot get the expected reward.” The third item is reverse coded, alpha = 0.81.

The order of the temporal discounting task and the optimism measure was counterbalanced.

### Results

#### Manipulation Check

To test whether participants’ sense of power was successfully manipulated, two independent coders who were blind to experimental hypotheses evaluated participants’ written essays in terms of how much power they expressed in the essays with a 5-point Likert scale (1 = low sense of power to 5 = high sense of power). The inter-rater reliability was high (*r* = 0.81, *p* < 0.01). Results from an independent-samples *t*-test showed that compared with the participants in the *low power* condition who were asked to recall an experience in which others had power over them (*M* = 2.60, *SD* = 0.99), the participants in the *high power* condition who were asked to recall an experience in which they had power over others (*M* = 3.42, *SD* = 1.01) were evaluated to have significantly more power (*t*(76) = 3.62, *p* < 0.01, Cohen’s *d* = 0.76, 95% CI = [1.27, 1.37]). Therefore, the power manipulation was effective in this study.

#### Descriptive Statistics

Consistent with hypotheses, temporal discounting was shown to be negatively correlated with sense of power (*r* = -0.24, *p* < 0.05), and with the level of optimism (*r* = -0.41, *p* < 0.001). In addition, there was a positive correlation between sense of power and the level of optimism (*r* = 0.27, *p* < 0.05). Gender and age were not associated with temporal discounting (refer to **Table 1**).

**Table 1:** Means, standard deviations, and bivariate correlations between study variables in Study 1 (*N* = 78).

Variables	*M*	*SD*	1	2	3
1 Age	20.44	0.98			
2 Rated power	3.25	0.63	0.08		
3 Optimism	3.42	1.33	-0.16	0.27^∗^	
4 Temporal discounting	0.69	0.27	0.17	-0.24^∗^	-0.41^∗∗∗^

*^∗^*p* < 0.05, ^∗∗^*p* < 0.01, ^∗∗∗^*p* < 0.001*.

#### The Influence of Power on Temporal Discounting

An independent sample *t*-test illustrated that the rate of temporal discounting for *high power* participants (*M* = 0.63, *SD* = 0.28) were significantly lower than the rate of temporal discounting for *low power* participants (*M* = 0.75, *SD* = 0.24), *t*(76) = 2.15, *p* < 0.05, Cohen’s *d* = 0.48, 95% CI = [0.01, 0.24]. We also conducted hierarchical regression analysis (**Table 2**). Model 3 indicated that power significantly reduced temporal discounting (β = -0.24, *p* < 0.05, 95% CI = [-0.25, -0.01]). After controlling gender and age (both non-significant), the influence of power on temporal discounting remained significant (β = -0.25, *p* < 0.05, 95% CI = [-0.25, -0.01]). Therefore, H1 was supported by the data (refer to the Appendix 1 for mediation analyses).

**Table 2:** Hierarchical regression analysis: the mediating effect of optimism on the relationship between power and temporal discounting in Study 1.

Variables	Level of optimism		Temporal discounting		
	Model 1	Model 2	Model 3	Model 4	Model 5
High Power	0.27*	0.28*	-0.24*	-0.25*	-0.15
Gender		-0.05		0.06	0.04
Age		-0.19		0.19	0.13
Optimism					-0.34**
*R*^2^	0.08	0.11	0.06	0.09	0.20
Δ*R*^2^	0.08*	0.04	0.06*	0.04	0.11**

*^∗^*p* < 0.05, ^∗∗^*p* < 0.01, ^∗∗∗^*p* < 0.001*.

## Study 2: The Influence of Power and Frustration Experience on Temporal Discounting Among Corporate Employees

To further establish the relationship between power and temporal discounting in a naturalistic setting and explore some mechanism behind the relationship, we conducted a survey study with a non-student population. In particular, we are testing hypotheses **H3a** and **H3b** while conceptually replicating **H1** and **H2**, investigating how frustration experience will affect the relationship between power and optimism, which influences temporal discounting. Study 2 was conducted among corporate employees in the Yangtze River Delta. Compared with university students in Study 1, corporate employees have richer personal experiences and more distinct background. Therefore, we expect to find more variations in personal frustration experience and the sense of power without experimental manipulation.

### Method

#### Participants

Participants were corporate employees from the Yangtze River Delta. Four hundred questionnaires were distributed to the sample population and 337 of them were returned (return rate = 84.25%). Among these returned questionnaires, 18 participants were dropped for completing less than half of the questionnaire. In addition, we excluded 14 participants for getting more than two indifference points in the temporal discounting task; and 31 participants for having a temporal discounting rate of less than 1 (the same exclusion criteria as in Study 1)^4^. After excluding these 64 participants from the sample, the number of valid questionnaires was 273 (valid rate 81.01%).

In the final sample, 52.01% (*n* = 142) were female, while 47.99% (*n* = 131) were male. The average age of participants was 30.89 years (*SD* = 7.02), ranging from 18 to 65. Most of the participants achieved a college or university diploma (*n* = 243, 89.01%)^5^.

#### Design and Procedure

Participants were sent a link of a survey questionnaire, which was comprised of measures on power, personal frustration experience, temporal discounting, optimism, and demographic information such as gender, age, education, occupation, and annual income.

##### The sense of power

Power was assessed with the generalized version of the Sense of Power Scale adapted from Anderson and Galinsky (2006). Participants were asked to rate their agreement with eight items such as “In my relationships with other, I think I have a great deal of power,” on a scale from 1 (“Strongly disagree”) to 5(“Strongly agree”). Among these items, the second, the fourth, the sixth and the seventh were reverse coded. The scale showed high internal consistency, alpha = 0.85. Prior research suggested that power assessed by the generalized version of the Sense of Power Scale is considered as trait power which is more stable in time and is influenced by personality and personal experiences (DeCelles et al., 2012).

##### Frustration experience

Frustration experience was measured by the Chinese version of the Life Events Scale (LES; Zhang et al., 1987), which was adapted from Holmes and Rahe’s (1967) social readjustment rating scale (the r with LES is 0.80). LES was consisted of 65 items, which included both negative and positive life events. Participants were asked to recall whether they had encountered those 65 events and write down the dates when the events occurred. The reason for writing down the dates was to facilitate memory retrieval and to improve the validity of the questionnaire by avoiding inattentive answering from participants. Since frustration experience was a focus in Study 2, only the ratings of negative life events were recorded. Ratings for each item were normalized for analysis.

##### Temporal discounting task (Hardisty and Weber, 2009)

In order to improve the external and ecological validity, an air gain scenario was adopted, which has high relevance for organizational employees in the industrial area where our participants were recruited. Participants were told to imagine that “the local county government was considering a temporary change to its emissions policy to study the effects of air quality on human health and the local wildlife. The particulate output of nearby factories would be immediately reduced for a period of 3 weeks, after which time the air quality would return to its former level, but the government was also considering making the change 1 year in the future, for a different length of time.” Participants were asked to consider only their personal preference (for improved air quality immediately or certain days in the future) as they made their choices, such as “Improved air quality immediately for 21 days, or improved air quality 1 year from now for 35 days.” Then the indifferent point was obtained and the temporal discounting rate of participants was calculated.

##### Optimism measure

The optimism measure was the same as in Study 1 (adapted from Anderson and Galinsky, 2006). Participants answered three items on a 7-point Liker scale (1 = disagree strongly to 7 = agree strongly), including ‘Although the limiting emission of pollutants will cause the loss of production, I believe the government has the ability to deal with it,’ ‘I am optimistic that the quality of air will improve in 1 year,’ and “I do not think that there is an unexpected situation where the quality of air cannot improve to the expected level.” The third item is reverse coded, alpha = 0.80.

### Results

#### Descriptive Statistics

Consistent with H1, **Table 3** showed a significant negative correlation between power and temporal discounting (*r* = -0.14, *p* < 0.05). We also found a significant negative correlation between frustration experience and optimism (*r* = -0.39, *p* < 0.01), and a significant positive correlation between power and optimism (*r* = 0.26, *p* < 0.01), which were consistent with the prediction that power and frustration experience were both associated with the level of optimism. In addition, there was a significant negative correlation between optimism and temporal discounting (*r* = -0.41, *p* < 0.01), and between temporal discounting and frustration experience (*r* = 0.41, *p* < 0.01), which supported our hypotheses as well.

**Table 3:** Means, standard deviations, and bivariate correlations between variables in Study 2 (*N* = 273).

Variables	*M*	*SD*	1	2	3	4	5	6
1 Age	30.89	7.02						
2 Education	2.81	0.62	-0.10					
3 Income	2.00	0.73	0.27^∗∗^	0.19^∗∗^				
4 Power	2.59	0.62	0.03	0.19^∗∗^	0.20^∗∗^			
5 Frustration	174.08	80.22	0.04	0.10	0.10	0.10		
6 Optimism	4.66	1.09	0.09	0.03	0.09	0.26^∗∗^	-0.39^∗∗^
7 Temporal discounting	0.39	0.16	0.04	0.10	-0.10	-0.14^∗^	0.41^∗∗^	-0.41^∗∗^

*^∗^*p* < 0.05, ^∗∗^*p* < 0.01, ^∗∗∗^*p* < 0.001. Level of education, 1 = equal to or below high school (technical secondary school) students, 2 = junior college students, 3 = graduate students, 4 = equal to or above postgraduate students; Annual income, 1 = below ¥40,000, 2 = between ¥40,000 and ¥80,000, 3 = above ¥80,000*.

#### Hierarchical Regression Analysis

In Study 2, frustration experience moderated the relationship between power and optimism. In other words, the interaction between power and frustration experience influenced temporal discounting via the level of optimism. **Table 4** showed that the interaction between power and frustration experience significantly influence the level of optimism (β = -0.15, *p* < 0.01, 95% CI = [-0.005, -0.001]) in model 3. Specifically, frustration experience negatively moderated the positive relationship between power and optimism. Therefore, H3a was supported by the data. In model 6, there was a significant interaction effect between power and frustration experience on temporal discounting (β = 0.12, *t*(265) = 2.13, *p* < 0.05, 95% CI = [0.000, 0.001]). In model 7, the level of optimism was included as a mediator and the results showed that the interaction effect between power and frustration experience turned non-significant (β = 0.08, *t*(264) = 1.48, *p* > 0.05) while the level of optimism remained a significant predictor for temporal discounting (β = -0.26, *t*(264) = -0.26, *p* < 0.001, 95% CI = [-0.055, -0.020]). Therefore, frustration experience moderated the relationship between power and temporal discounting via the level of optimism, and H3b was supported by the data.

**Table 4:** Analysis of the moderating effect of frustration experience on the relationship between power, optimism, and temporal discounting in Study 2.

Variables	The level of optimism			Temporal discounting			
	Model 1	Model 2	Model 3	Model 4	Model 5	Model 6	Model 7
Gender	0.03	0.06	0.06	0.06	0.02	0.02	0.04
Age	0.08	0.11	0.11	0.10	0.07	0.07	0.10
Education	0.03	0.03	0.03	0.14*	0.12*	0.12*	0.13*
Income	0.06	-0.00	0.01	-0.15*	-0.10	-0.11	-0.11
Power		0.30***	0.29***		-0.19**	-0.18**	-0.11
Frustration		-0.43***	-0.41***		0.42***	0.39***	0.29***
Power × Frustration			-0.15**			0.12*	0.08
Optimism							0.26***
*R*^2^	0.01	0.26	0.28	0.03	0.22	0.24	0.29
Δ*R*^2^	0.01	0.24***	0.02**	0.03	0.19***	0.01*	0.05***

*^∗^*p* < 0.05, ^∗∗^*p* < 0.01, ^∗∗∗^*p* < 0.001*.

**FIGURE 1:**
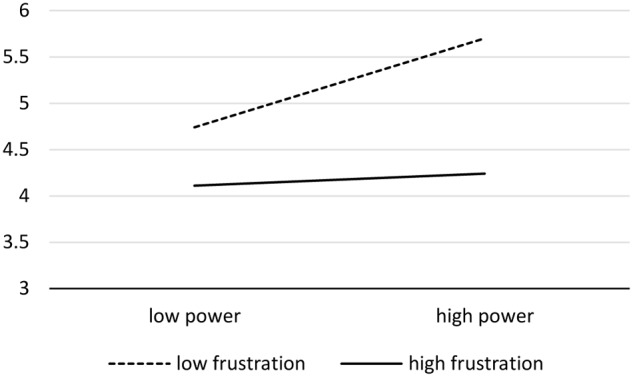
The moderating effect of frustration experience on the relationship between power and the level of optimism.

In order to explain the moderating effect of frustration experience on the relationship between power and optimism, a simple slope analysis (Aiken et al., 1991) was conducted. We divided the participants into low frustration group (1 SD below the average) and high frustration group (1 SD above the average). **Figure 1** presents the results from the single slope analysis.

In the low frustration group, power positively predicted the level of optimism (β = 0.44, *t*(49) = 3.39, *p* < 0.001, 95% CI = [0.28, 1.10]), whereas power did not predict the level of optimism in the high frustration experience group (β = 0.04, *t*(51) = 0.30, *p* > 0.05). Therefore, frustration experience negatively moderated the positive relationship between power and optimism and H3a was confirmed (refer to the Appendix 1 for mediation analyses).

## Study 3: Power and Temporal Discounting Across Ethnic Groups – the Effect of *Danbo* (Indifference to Fame and Wealth)

Study 3 investigated the influence of *Danbo* trait on the relationship between power and temporal discounting with a 2 (ethnicity) × 2 (power) independent factorial design. In order to capture the variability of *Danbo* trait, we recruited a sample from the ethnic Tibetan group, who are known to have high *Danbo* trait due to their religious practices (Wang and Cui, 2004), along with a sample of Han students (the majority of Chinese people are identified as ethnic Han). We used the same procedure as in Study 1 to experimentally manipulate sense of power. *Danbo* trait and Social Desirability of participants were measured afterward. In order to prevent participants from guessing the experimental purposes^6^, the scale of *Danbo* trait and Social Desirability of participants were integrated into a scale along with filler questions. In addition, participants were asked to complete a time discounting task and rating the level of decision optimism.

### Method

#### Participants

Study 3 recruited 105 participants, including 50 subjects from Han ethnic group, and 55 from Tibetan ethnic group^7^. Ten Han subjects were excluded from the data (four did not complete experimental manipulation; two had two or more indifference points in temporal discounting; and four had a zero time discounting rate). Fifteen subjects from the Tibetan group had to be excluded from the same criteria (eight subjects did not complete the recall task; five had two or more indifference points and two had a zero time discounting rate in the time discounting task)^8^.

For the final sample of Han participants, there were 9 men (22.5%) and 31 women (77.5%), with an average age of 21.13 years old (*SD* = 1.88) ranging from 19 to 25. For Tibetan participants, there were 16 men (40%) and 24 women (60%), with an average age of 20.00 years old (*SD* = 0.75) ranging from 19 to 23.

#### Design and Procedure

##### Power mind-set manipulation

Participants were randomly assigned into a *high powerI* condition and *low power* condition. Manipulation was the same as in study 1.

*Danbo* trait measure. *Danbo* was assessed by Chinese personality scale of *Danbo* trait edited by Wang and Cui (2004). This scale has six items, for example, “I think it would be good to be an ordinary person” and “I am eager to gain more achievements,” with a 5-point Likert scale (1 = strongly disagree; 5 = strongly agree). Three items were reverse coded. The Cronbach’s α of the scale is 0.72 for the current study.

##### Temporal discounting task

We used the same scenario to calculate participants’ tendency of temporal discounting as in Study 1.

##### Optimism measure

The level of optimism was assessed by the same materials as in Study 1. The order temporal discounting task and optimism measure was counterbalanced.

##### Social desirability

The control variable of social desirability was measured from the Balanced Inventory of Desirable Response, along with demographic questions (BIDR; Paulhus, 1988). There were 20 items, such as “I sometimes suffers a loss because of hesitation,” “I never throw rubbish in the street,” with a 5-point Likert scale (1 = strongly disagree; 5 = strongly agree). Half of the items in the scale were reverse coded. The Cronbach’s α of the scale is 0.65 for the current study.

### Results

#### Manipulation Check

As in Study 1, to test whether participants’ sense of power was successfully manipulated, two independent coders who were blind to experimental hypotheses evaluated participants’ written essays in terms of how much power they expressed in the essays with a 5-point Likert scale (1 = low sense of power to 5 = high sense of power). The inter-rater reliability was high (*r* = 0.78, *p* < 0.01). Results from an independent-samples *t*-test showed that compared with *low power* participants who were asked to recall an experience in which others had power over them (*M* = 1.99, *SD* = 0.70), *high power* participants who were asked to recall an experience in which they had power over others (*M* = 3.28, *SD* = 0.88) were evaluated to have more power (*t*(78) = -7.24, *p* < 0.001, Cohen’s *d* = 1.72, 95% CI = [-1.65, -0.94]). Therefore, the power manipulation was effective in this study.

#### Descriptive Statistics and Comparative Analysis

As shown in **Table 5**, the level of optimism was positively correlated with power (*r* = 0.39, *p* < 0.01), and negatively correlated with temporal discounting (*r* = -0.40, *p* < 0.01).

**Table 5:** Descriptive statistics in Study 3 (*N* = 80).

Variables	*M*	*SD*	2	4	5	6	7
2 Age	20.59	1.54					
4 Social desirability	3.12	0.39	-0.01				
5 Rated Power	2.65	1.03	-0.06	-0.17			
6 *Danbo*	2.95	0.69	-0.10	0.17	-0.06		
7 Optimism	3.91	1.13	-0.10	0.13	0.39^∗∗^	0.03	
8 Temporal discounting	0.64	0.51	0.22	-0.13	-0.21	-0.23^∗^	-0.40^∗∗^

*^∗^*p* < 0.05, ^∗∗^*p* < 0.01*.

We conducted independent *t*-tests to examine the comparative differences between participants from Han and Tibetan ethnic groups on the key experimental variables. Tibetan students reported significantly higher *Danbo* (*M* = 3.23, *SD* = 0.76) than Han students (*M* = 2.68, *SD* = 0.56), *t*(78) = 3.79, *p* < 0.001, Cohen’s *d* = 0.86, 95% CI = [0.26, 0.83]. In addition, Tibetan student reported higher level of optimism (*M* = 4.18, *SD* = 1.29) than Han students (*M* = 3.63, *SD* = 0.89), *t*(78) = 2.22, *p* < 0.05, Cohen’s *d* = 0.50, 95% CI = [0.06, 1.04]. There was no difference in social desirability between Han (*M* = 3.06, *S*D = 0.41) and Tibetan students (*M* = 3.18, *SD* = 0.37), *t*(78) = 1.38, *p* > 0.05.

#### The Difference of the Relationship between Power and Temporal Discounting among Han and Tibetan Subjects

Since the *Danbo* of Han subjects and Tibetan subjects was significantly different, we analyzed the Han subjects and Tibetan subjects discretely. **Table 6** showed the relationship between power and temporal discounting for Han subjects. Model 3 indicated that power was negatively related to temporal discounting (β ((-0.34, t(38) (-2.25, *p* < 0.05, 95% CI = [-0.72, -0.04]). After including the social desirability variable, the relationship between power and temporal discounting remained significant.

**Table 6:** Results from regression models for Han subjects in Study 3.

Variables	Optimism		Temporal discounting		
	Model 1	Model 2	Model 3	Model 4	Model 5
High Power	0.38^∗^	0.38^∗^	-0.34^∗^	-0.34^∗^	-0.17
Social desirability		-0.05		-0.11	-0.14
Optimism					-0.45^∗∗^
*R*^2^	0.15	0.15	0.12	0.13	0.30
Δ*R*^2^	0.15^∗^	0.00	0.12^∗^	0.01	0.17^∗∗^

*^∗^*p* < 0.05, ^∗∗^*p* < 0.01*.

**Table 7** showed the relationship between power and temporal discounting for Tibetan subjects. Different from the Han subjects, Model 3 suggested that there was no significant relationship between power and time discounting (β = -0.03, *p* > 0.05). In other words, for Tibetans, differences in the sense of power would not predict their tendency of time discounting (refer to the Appendix 1 for mediation analyses).

**Table 7:** Results from regression models for Tibetan subjects in Study 3.

Variables	Optimism		Temporal discounting		
	Model 1	Model 2	Model 3	Model 4	Model 5
High Power	0.41^∗∗^	0.48^∗∗^	-0.03	-0.01	0.13
Social desirability		0.33^∗^		0.06	0.15
Optimism					-0.29
*R*^2^	0.17	0.27	0.00	0.01	0.07
Δ*R*^2^	0.17^∗∗^	0.10^∗^	0.00	0.00	0.06

*^∗^*p* < 0.05, ^∗∗^*p* < 0.01, ^∗∗∗^*p* < 0.001*.

## Discussion

### Analysis for the Results

Three studies explore the relationship between power and temporal discounting. Study 1 investigates the relationship between power and temporal discounting and the underlying mechanism. The results present a lower tendency of temporal discounting among individuals with high sense of power, compared to those with low sense of power. This result is in line with the findings of Joshi and Fast (2013), which found that individuals with a high sense of power saved more than those with low sense of power in general. In other words, it is more likely for people with high sense of power to choose savings or investment (delayed gratification) over immediate consumption. It is also consistent with prior findings that while the construal level theory predicts temporal discounting, power increases people’s focus on the high construal level attributes in the future rather than the low construal level attributes in the present (Trope and Liberman, 2003, 2010; Magee and Smith, 2013).

Study 2 is a conceptual replication regarding the results of Study 1 in a real organizational setting, and progressively investigates the boundary conditions by introducing frustration experience. The relationship between power and the level of optimism has been moderated by frustration experience. A high level of frustration experience is found to weaken the positive effect of optimism. Power and frustration experience jointly influence the level of optimism, which subsequently influences individuals’ tendency of temporal discounting. This finding is consistent with Lammers et al. (2008), which noted that when power was threatened, high power individuals would engage in more avoid behaviors than approach behaviors. Study 2 indicated that when high power individuals remembered more frustration experiences, their levels of optimism may decrease, leading to higher likelihood of choosing immediate rewards rather than delayed rewards. Maner et al. (2007) also found that when positioned in an unstable organizational hierarchy, people with high sense of power were more likely to make conservative rather than risky decisions. The instability of the organizational hierarchy implies higher probability of losing power at any time; thus even the powerful people are eager for immediate small rewards instead of larger rewards in a distant future.

Study 3 investigates the impact of *Danbo* trait, a unique cultural feature referring to indifference of fame and wealth, on the mediating effect of optimism. It was found that in the case of low *Danbo* trait, optimism mediated the relationship between power and time discounting; however, in the case of high *Danbo* trait, the mediating effect of optimism on the relationship between power and time discounting was not significant. Although research did not directly test the causal relationship between *Danbo* and time discounting, previous studies offer some insights. Wang et al. (2011) found that greedy individuals (low *Danbo* trait) showed more self-interested behavior, and lower ability to resist the temptation of getting an instant reward. For a low power individual with high *Danbo* trait, even when her level of optimism level is low, she may have lower tendency of time discounting. In addition, Metcalfe and Mischel (1999) find individuals who have less material desire prefer cool rather than hot system when making decision, and pay more attention to the long-term interests rather than short-term benefits (Wang et al., 2013). These findings offer theoretical implications why *Danbo* can affect the causal chain of power and temporal discounting.

### Implication

Firstly, this study expanded the research on factors influencing temporal discounting. From investigating characteristics of the decision maker, such as power, optimism, and Danbo, we enriched our understanding on factors that may affect intertemporal choices and time preference. Secondly, while power has been conceptualized as a psychological variable (Study 1) and structural variable (Study 2) respectively, we are able to reach a consistent conclusion that power reduces temporal discounting in general. The current research promoted external validity of the results and improved our understanding on the nature of the relationship of power and temporal discounting. Moreover, we conceptualized temporal discounting in a behavioral framework, and analyzed individuals’ approach and avoid motives when they are optimistic, frustrated, or express high or low Danbo trait, extending the approach-avoidance behavioral approach.

In daily life and work, individuals are faced with intertemporal choices frequently, ranging from shopping to money investment and management, and life-time plans. During the process of intertemporal choice, individuals, however, showed the tendency of temporal discounting, pursuing near-future rewards while devaluing distant-future rewards. It is thus important for individuals to be aware of such tendency, in order to make more wise decisions. In the instances where reducing temporal discounting is necessary, we offer several humble suggestions based on the results of the current study: (1) Before making intertemporal choices, recall situations in which one has power or sense of control; (2) Keep optimistic and confident while making intertemporal choices; (3) Avoid to make intertemporal choices when one is experiencing frustration. At the same time, this study provided some implications for organization management: (1) When making decisions like the long-term planning of the organization, which call for the weighing of pros and cons of the near-future rewards and the distant-future development, leaders or powerful individuals should be present at the decision making processes; (2) Leaders’ personal experiences are likely to influence the rationality of their decisions. Therefore, decisions concerning the development of the organization, which are made by leaders who are experiencing great frustration, should be considered carefully. (3) Individuals with high *Danbo* trait may be less biased when considering the future rewards, and it is worthwhile to pay more attention to their recommendations on intertemporal choice. All of these comments and suggestions could be possibly developed in future research.

There are limitations of the present research that we would like to improve in the future. In Study 2, power was self-reported as a trait construct from a heterogeneous sample, which made the reported effect size uncomparable with those from Studies 1 and 3, where power was experimentally manipulated. Further studies should replicate the results with different measures of the current theoretical constructs and across diverse populations. Study 3 used Tibetan and Han students mainly to distinguish *Danbo* qualities, while there might be noise variables that are different between Han and Tibetan ethnic groups, which made it hard to attribute the difference to *Danbo* only. However, we merit the finding that the relationship between power and temporal discounting manifests non-uniformly across different cultural groups, which reinforces the importance of considering the characteristics of decision makers in intertemporal choice.

## Conclusion

The current studies investigated the effects of power on temporal discounting. The results indicated that sense of power reduces temporal discounting, possibly through a heightened level of optimism in high-power individuals, and there exists boundary conditions where individual differences of frustration experience and *Danbo* trait (indifference to fame and wealth) can affect the relationship between power and temporal discounting. The current research contributes to our understanding of intertemporal choice and decision making by focusing on the individual and cross-cultural differences of the decision makers who are facing intertemporal choices.

## Ethics Statement

This study was carried out in accordance with the recommendations of Soochow University’s Committee for Research with Human and Animal Subjects with written informed consent from all subjects. All subjects gave written informed consent in accordance with the Declaration of Helsinki. The protocol was approved by the Committee for Research with Human and Animal Subjects of Soochow University, China.

## Author Contributions

JD and SW contributed to the hypotheses initiation and experimental design. JD and LS collected all the experimental data. All authors took part in data analysis and manuscript writing.

## Conflict of Interest Statement

The authors declare that the research was conducted in the absence of any commercial or financial relationships that could be construed as a potential conflict of interest.

## Footnotes

1 However, as we do not experimentally manipulate optimism, we are less confident in the inferences drawn from traditional mediation analyses as the estimates from an average indirect effect can be biased (Bullock et al., 2010).

2 Normally there exists only one indifference point in temporal discounting within a fixed time frame. In the current study, if the future monetary amount is below a participant’s indifference point, she will choose an instant reward of ¥120; if the choice amount is above her indifference point, she is willing to wait to take more money.

3 Including these outliers did not change the results.

4 Including these participants did not change the results.

5 There were 10 participants whose education level was equal to or lower than technical secondary school (3.66%); 20 participants were postgraduate students (7.33%).

6 No participant expressed suspicion or guessed our hypotheses upon debriefing.

7 The Han subjects were recruited from Soochow University while the Tibetan subjects were recruited from Lhasa Normal University in Tibet.

8 Including the “irrational” participants did not change the final results.
