# Supplementary material for: Do the Powerful Discount the Future Less? The Effects of Power on Temporal Discounting
Source: Front Psychol. 2017 Jun 21;8:1007. doi: 10.3389/fpsyg.2017.01007 (PMC5479056; doi:10.3389/fpsyg.2017.01007)
Supplement: Supplementary file 1 [file Data_Sheet_1.docx]

**Appendix 1: The mediating effect of optimism in the relationship between power and temporal discounting**

1. Study 1

According to model 2 in Table 2, after controlling gender and age (both non-significant), power had a significant influence on the level of optimism (*β*=0.28, t(76)=2.56, *p*<0.05, 95%CI=[0.17, 1.34]). In addition, H1 has been confirmed that power had a significant influence on temporal discounting, our main dependent variable. In model 5, the mediator, the level of optimism, was included into the regression model and the results showed that the coefficient of manipulated power turned non-significant (*β*=-0.15，*p*>0.05) while the coefficient of the level of optimism remained significant (*β*=-0.34，t(76)=-3.09,*p*<0.01,95%CI=[-0.11,-0.02]). Thus the level of optimism fully mediated the relationship between power and temporal discounting. H2 was supported by the data as well.

According to the approach of bootstrap proposed by Edwards and Lambert (2007) and the software developed by Hayes (2013), controlled variables (gender and age), an independent variable (manipulated power), a mediator (the level of optimism) and a dependent variable (temporal discounting) was included in this model. If the bootstrapped confidence interval for the indirect effect through the proposed mediator is outside of zero, the mediating effect can be regarded as being significant (Preacher &Hayes, 2008). In the model of this study, the 95% confidence interval for level of optimism is from -.12 to -.01. Therefore, the mediating effect of optimism was significant.

In conclusion, we find that individual level of optimism mediated the relationship between power and temporal discounting. High power individuals are more optimistic when evaluating risks in intertemporal choices (Levine, 2010), and their risk compensation is also lower, so they are less likely to experience temporal discounting.

1. Study 2

The control variables (gender, age, level of education, and annual income), the independent variable (power), the mediator (level of optimism), the moderator (frustration experience), the dependent variable (temporal discounting) was included in the bootstrapping model. The 95% confidence interval for the level of optimism is from -.07 to -.02. Therefore, the mediated moderation model was testified.

1. Study 3

Table 6 showed the level of optimism mediated the relationship between power and temporal discounting for Han subjects. Model 1 and Model 3 indicated that power was positively related to optimism (β = 0.38, t(38)=2.54, p <0.05, 95%CI=[0.14, 1.20]) and negatively related to time discounting (β = -0.34, t(38)=-2.25, p <0.05, 95%CI=[-0.72, -0.04]). After including the mediating variable, optimism, the coefficient of power became non-significant (β = -0.17, t(38)=-1.16, p> 0.05) while the coefficient of optimism remained significant (β = -0.45, t(38)=-2.97, p <0. 01, 95%CI=[-0.48, -0.09]). This indicates that for the Han subjects, optimism mediated the relationship between power and temporal discounting.

Table 7 showed the level of optimism mediated the relationship between power and temporal discounting for Tibetan subjects. Model 1 indicated that there was a significant positive relationship between power and optimism (β = 0.41, t(38)=2.79, p <0.01, 95%CI=[0.29, 1.81]), similar to the Han subjects. Interestingly, different from the Han subjects, Model 3 suggested that there was no significant relationship between power and time discounting (β = -0.03, p> 0.05). In other words, for Tibetans, differences in the sense of power would not predict their tendency of time discounting. And the level of optimism no longer served as a significant mediator.

We modeled the control variables (gender, age, and social desirability), the independent variable (power), the mediating variables (the level of optimism), the moderating variable (*Danbo* trait), and the dependent variable (temporal discounting) by bootstrap. It was found that when the *Danbo* trait was low (for Han subjects), the mediating effect of optimism was significant (95% confidence interval for optimism [-0.59, -0.09]); when *Danbo* trait was high (for Tibetan subjects) the mediating effect of optimism was not significant (95% confidence interval for optimism [-0.07,0.04]). This supported a moderated mediated model, which was consistent with hypothesis 4.

It was found that in the case of low *Danbo* trait, optimism mediated the relationship between power and time discounting; however, in the case of high *Danbo* trait, the mediating effect of optimism on the relationship between power and time discounting was not significant.
